# Expression of concern: One-pot sol–gel synthesis of reduced graphene oxide uniformly decorated zinc oxide nanoparticles in starch environment for highly efficient photodegradation of methylene blue

**DOI:** 10.1039/c8ra90021c

**Published:** 2018-03-14

**Authors:** Andrew Shore

**Affiliations:** Royal Society of Chemistry Thomas Graham House, Science Park, Milton Road Cambridge UK CB4 0WF advances-rsc@rsc.org

## Abstract

Expression of concern for ‘One-pot sol–gel synthesis of reduced graphene oxide uniformly decorated zinc oxide nanoparticles in starch environment for highly efficient photodegradation of methylene blue’ by Majid Azarang *et al.*, *RSC Adv.*, 2015, **5**, 21888–21896.

The following article ‘One-pot sol–gel synthesis of reduced graphene oxide uniformly decorated zinc oxide nanoparticles in starch environment for highly efficient photodegradation of methylene blue’ by Majid Azarang*^ab^, Ahmad Shuhaimi^a^, Ramin Yousefi^c^ and Siamak Pilban Jahromi^a^ has been published in *RSC Advances*.

*RSC Advances* is publishing this expression of concern in order to alert our readers to the fact that we are presently unable to confirm the accuracy of the data reported in Fig. 2(a, b) and 5 of this *RSC Advances* paper.

We have contacted the University of Malaya to request an investigation into the validity of the published figures. We understand that an investigative process is on-going at the University of Malaya, and this notice will be updated when a final outcome is reached.

Andrew Shore

2nd March 2018

Executive Editor, *RSC Advances*

## Supplementary Material

